# Engaging leadership and work recovery among key personnel of a major health-care and social services reform

**DOI:** 10.1108/LHS-09-2024-0109

**Published:** 2025-04-16

**Authors:** Kirsikka Selander, Nina Nevanperä, Risto Nikunlaakso, Eveliina Korkiakangas, Jaana Laitinen

**Affiliations:** Finnish Institute of Occupational Health, Kuopio, Finland; Finnish Institute of Occupational Health, Helsinki, Finland; Finnish Institute of Occupational Health, Oulu, Finland

**Keywords:** Work recovery, Engaging leadership, Job demands, Job well-being, Organizational change, Health care and social services

## Abstract

**Purpose:**

The purpose of this paper is to investigate the experiences of engaging leadership and work recovery among preparers of a major health-care and social services reform in Finland in 2022; to investigate whether engaging leadership was associated with work recovery; and to investigate whether engaging leadership alleviated the harmful effect of job demands on work recovery.

**Design/methodology/approach:**

Altogether 258 reform preparers participated in four job well-being surveys. Means and paired *t*-test were used to measure engaging leadership and work recovery during the study period. Hierarchical multiple linear regression analysis was used to analyze associations between engaging leadership, job demands and recovery from work.

**Findings:**

Experiences of engaging leadership and recovery from work decreased during the study period. A change in engaging leadership had a small positive association with work recovery at endpoint. A change in job demands had a stronger association with work recovery at endpoint. Engaging leadership did not alleviate the association between job demands and recovery from work.

**Originality/value:**

This study expands previous work recovery literature by demonstrating that engaging leadership style can improve work recovery during health and social care reforms. However, engaging leadership style alone is insufficient to alleviate job demands, and therefore more effective management of job demands is needed. Practically, the findings can be used to plan and lead future reforms.

## Introduction

Organizing health-care and social services and ensuring sufficiency of workforce is a common challenge in many countries (de Matos *et al.*, 2024). Moreover, aging population further increases the need for care and services, necessitating even larger workforce (Zhang *et al.*, 2023; Cristea *et al.*, 2020). To solve the challenge, Finland implemented a significant administrative reform in public health-care and social services in the beginning of 2023. The reform aimed to ensure equal services, reduce inequalities in health and well-being and reduce health-care and social services costs. During the reform, the responsibility for organizing health-care, social and rescue services were transferred from 306 municipalities (primary health-care and social services) and 20 hospital districts (specialized medical treatment) to 21 well-being services counties established at the beginning of the reform (Ministry of Social Affairs and Health, 2023). The changes were implemented rapidly, in approximately one and a half years.

The preparation of the reform was done by health-care and social service specialists and managers who performed the preparation tasks alongside their normal duties at hospital districts and municipalities. The preparation time was hectic, as health-care and social service organizations were still dealing with the impact of the COVID-19 pandemic. Previous research suggest that work-related mental strain is more detrimental to work recovery than physical strain (Winwood and Lushington, 2006). Therefore, the intense pace of preparation and the overwhelming workload strained the preparers (Laitinen *et al.*, 2023), potentially increasing the risk of poor work recovery. As health-care and social services organizations across Western countries persistently encounter constant pressures for organizational changes (de Matos *et al.*, 2024), it is crucial to glean insights from these experiences.

Organizational changes have been shown to negatively affect work characteristics such as heightened uncertainty, unclear work roles and increased job demands (Kivimäki *et al.*, 2001; Oreg *et al.*, 2011; Smollan, 2017). These changes further prevent work recovery, which is associated with occupational well-being and job performance (Binnewies *et al.*, 2010; Kinnunen and Feldt, 2013; de Bloom *et al.*, 2015; Steed *et al.*, 2021). Poor recovery, on the other hand, can pose health risks and increase the risk for occupational burnout (Gluschkoff *et al.*, 2016; Geurts and Sonnentag, 2006; Kivimäki *et al.*, 2006). To avoid these consequences, more attention should be placed on work recovery and ways to support it.

Work recovery means that energy levels and resources are replenished and the psychophysiological systems return to their baseline level (Sonnentag *et al.*, 2017; Sonnentag *et al.*, 2022). Recovery may occur during the workday, such as during breaks and microbreaks, or during leisure time in the evenings and between work shifts and vacations (Sonnentag *et al.*, 2022; Virtanen *et al.*, 2021). Engaging in recovery-enhancing activities during breaks and leisure time, such as psychological detachment from work, physical exercise and sufficient sleep, has been highlighted as particularly important for facilitating work recovery (Sonnentag, 2018). However, as job strain and demands increase during organizational changes (Kivimäki *et al.*, 2001; Greubel and Kecklund, 2011; Oreg *et al.*, 2011), it becomes more challenging for individuals to detach from work and engage in recovery-enhancing activities (Rodriguez-Muñoz *et al.*, 2012; Bakker and de Vries, 2021). Therefore, particularly during organizational change, it is crucial for organizations to promote possibilities to recover from work.

Previous recovery literature has mainly concentrated on enhancing psychological recovery experiences, as well as the role of breaks and work scheduling in the recovery process (Verbeek *et al.*, 2019; Gifkins *et al.*, 2020; Virtanen *et al.*, 2021; Sonnentag *et al.*, 2022), with less emphasis on how managers can support recovery (Selander *et al.*, 2023; Czakert *et al.*, 2024). Nevertheless organizations, and particularly leaders who have regular contact with employees, have various possibilities to control the job strain employees are exposed to Schaufeli (2015) and to support recovery from work. For example, during organizational changes leaders have an important role in reducing employees’ uncertainty, clarifying work roles and alleviating job demands (Oreg *et al.*, 2011). Managing these stressors is important for the overall success of an organizational change (Peng *et al.*, 2021; Wiatr, 2022) and for the well-being of employees (Skakon *et al.*, 2010; Inceoglu *et al.*, 2018).

In this study, we focused on engaging leadership and its associations on work recovery. Engaging leaders influence their employees’ perceptions of the work to ensure that they can thrive in their roles and remain healthy and productive. They achieve this by allocating and managing the job strain experienced by team members (Schaufeli, 2015, 2021). To our knowledge, the impact of engaging leadership on the work recovery of preparers during organizational changes has not been previously studied, although preparers have a pivotal role in the success of health and social care reforms currently underway across Europe (de Matos *et al.*, 2024). Thus, our aim was to describe experiences of engaging leadership (Schaufeli, 2015) and work recovery during the preparation of the reform.

### Engaging leadership and work recovery

Engaging leadership has its root in self-determination theory, which suggest that employees stay healthy and motivated in the workplace when their basic psychological needs (autonomy, competence and relatedness) are met in a way that they are able to:

- decide about the tasks they have to perform (autonomy);
- use their skills (competence); and
- receive positive feedback from others at work (relatedness) (Van den Broeck *et al.*, 2008; Ryan and Deci, 2018; Ryan *et al.*, 2021; Schaufeli, 2021).

According to Schaufeli (2015), engaging leaders satisfy these basic psychological needs by facilitating (empowering), strengthening, inspiring and connecting employees.

By facilitating employees, such as supporting freedom and responsibility, employees feel free to make their own decisions, which in turn fosters feelings of autonomy and control over their own work. By giving employees more challenging tasks, stimulating employees’ skills and by giving positive feedback, employees feel more mastery and competent over work. By inspiring employees toward a shared goal leaders in turn create sense of vision, and by acknowledging each member’s contribution in this process they foster employees experience of the meaningful work. Connecting employees with other team-members, by encouraging team-level collaboration and creating team spirit in turn, creates sense of relatedness with others (Schaufeli, 2015, 2021).

Autonomy or mastery over work, feelings of competence, meaning and relatedness to others are also important determinants in the daily recovery process (Newman *et al.*, 2014; Virtanen *et al.*, 2021). To our knowledge, engaging leadership has been rarely studied in relation to work recovery (Selander *et al.*, 2023), but there is evidence suggesting that managers and leaders can facilitate the recovery of employees by empowering them and providing them sense of autonomy (Chan *et al.*, 2022). Thus, we assumed that:

*H1*. Engaging leadership has a direct positive effect on work recovery.

Furthermore, engaging leaders do not only shape their followers’ perceptions of the work environment, but they also effectively manage the job demands employees are exposed to (Schaufeli, 2015). Thus, we assumed that:

*H2*. Engaging leadership moderates the harmful effects of job demands on work recovery.

## Data and methods

The data consisted of key persons, such as leaders, managers and experts who participated in preparing the launch of Finnish well-being services counties (*n* = 258). The well-being of these preparers was monitored with monthly surveys between February and November 2022 (excluding July). Preparers from 20 preparation areas participated in the study. Only one preparation area did not participate. The number of respondents varied between 231 and 418 per month and the response rate varied between 21% and 37%. There were two types of monthly surveys: extensive (March, June, September and November) and brief (February, April, May, August and October).

The data of the extensive surveys was used in the analyses. Because the data of those who replied to all four questionnaires was small (*n* = 66), the data was collected by including those who answered to the questionnaires *first time* either March (*n* = 300) or June (*n* = 343), and *last time* either September (*n* = 348) or November (*n* = 342). To investigate the change over time, respondents who answered both, first time and last time, were included to the analyses (*n* = 258).

*Work recovery* was measured with one-item recovery scale developed by Kinnunen *et al.* (2011), who found that it exhibited similar characteristics to longer work recovery scales, such as need for recovery, reflecting comparable patterns of correlations with antecedents and consequences. Respondents were asked to assess whether they recover from the strain caused by the workday before the next day on a scale of 0 = not at all to 10 = completely, instead of the five-step response scale used in the original measurement.

*Engaging leadership* was measured using shortened version of Schaufeli’s (2015, 2021) engaging leadership scale. One item from each dimension, excluding inspiring leadership, was included. “My supervisor encourages team members to use their own strengths” describes strengthening leadership, “My supervisor actively encourages team members to aim for the same goals” describes connecting leadership and “My supervisor encourages team members to give their own opinion” describes empowering leadership. The response scale was a five-point scale from 1 = totally disagree to 5 = totally agree. Engaging leadership in the beginning (Cronbach’s alpha = 0.93) and in the end (Cronbach’s alpha = 0.92) of the study period was calculated as mean of these items and changes as subtraction between the end and beginning.

*Job demands* was measured with two items from the job content questionnaire (Karazek *et al.*, 1998): “I am required to do an unreasonable amount of work” and “I don’t have enough time to get my work done”. The response scale ranged from 1 = strongly agree to 5 = strongly disagree. Job demands in the beginning (Cronbach’s alpha = 0.85) and in the end (Cronbach’s alpha = 0.88) of the study period was calculated as mean of these items and changes as subtraction between the end and beginning.

As *control variables*, we used recovery at the beginning of the study period, sleep quality, gender (female/male), age and time between responses (in months). Sleep quality was controlled as it has been considered as a crucial part of the mental and physical recovery process (Åkerstedt *et al.*, 2009; Sonnentag, 2018). Furthermore, we added as a control variable whether respondent conducted his/her preparation tasks in a so-called “fragmented region”. In a fragmented region, health-care, social and rescue services were initially scattered across different municipalities and organizations and the coordination of the services in the beginning of the preparation started from scratch. In other regions, health-care and social services consortia or other forms of cooperation already existed before the preparation began.

### Statistical analysis

To describe experiences of engaging leadership and recovery from work across different background groups during the preparation year we used descriptive statistics (means and standard deviations) and paired *t*-test. Hierarchical multiple regression analysis was used to analyze whether engaging leadership style can promote work recovery. In the first step, we entered control variables and change in job demands. In second step, to test *H1*, we added change in engaging leadership. In third step, to test *H2*, we added interaction between change in job demands and engaging leadership. To avoid multicollinearity of the variables, we centered them before the analysis. IBM SPSS statistics version 27 was used in the analysis.

## Results

Most of preparers were women (78%) and on average 49 years old (SD = 9.7). About half of them worked in fragmented regions (46%). Engaging leadership and recovery from work decreased during the study period. Engaging leadership declined especially among women, those over 56 years old and those working in the fragmented regions. Recovery from work declined especially among women and youngest employees (Table 1).

**Table 1.** Means, standard deviations and paired *t*-test results for engaging leadership and recovery from work measured at the beginning and end of the study period

	*N*	Engaging leadership			Recovery from work		
		Beginning mean (SD)	End mean (SD)	*t*-test	Beginning mean (SD)	End mean (SD)	*t*-test
All	206	3.94 (0.99)	3.68 (1.04)	4.16***	6.68 (2.38)	6.35 (2.44)	2.45*
*Gender*							
Women	154	3.94 (0.94)	3.63 (1.05)	2.84**	6.85 (2.29)	6.35 (2.36)	3.22**
Men	53	3.97 (1.09)	3.86 (0.97)	0.57	6.46 (2.49)	6.44 (2.67)	0.04
*Age groups*							
21–45	81	3.86 (1.09)	3.51 (1.05)	2.01	6.26 (2.35)	5.55 (2.57)	2.79**
46–55	90	3.85 (0.99)	3.74 (1.05)	0.67	6.79 (2.41)	6.58 (2.41)	0.90
56-	81	4.06 (0.87)	3.72 (1.04)	2.06*	7.31 (2.06)	6.96 (2.22)	0.35
*Fragmented regions*							
Yes	153	3.94 (1.01)	3.58 (1.05)	3.54***	6.45 (2.50)	6.14 (2.47)	1.78
No	132	3.94 (0.98)	3.76 (1.02)	2.37*	6.86 (2.26)	6.53 (2.41)	1.72

**Note(s):** *p < 0.01, **p < 0.01, ***p < 0.001; the results are provided for the entire sample and separated by gender, age groups and fragmented regions

**Source(s):** Authors’ own work

### Association between engaging leadership and recovery from work

Bivariate correlations are presented in Table 2. Change in engaging leadership had a positive correlation with endpoint work recovery and negative correlation with change in job demands. Positive association between change in engaging leadership and work recovery remained statistically significant even after controlling for baseline recovery, sociodemographic factors and sleep quality, giving support to *H1*. The association between change in engaging leadership and work recovery was, however, relatively small, as it explained only two additional percentage points of the work recovery variance (adj. *R*^2^). A change in job demands had the strongest association with work recovery (Table 3). The interaction term of change in job demands and change in engaging leadership had a statistically insignificant effect, rejecting *H2*.

**Table 2.** Pearson correlation coefficients and their statistical significance (*p*-values)

	Recovery at the end	Recovery at the beginning	Change in sleep problems	Change in job demands	Change in engaging leadership
Recovery at the end	1				
Recovery at the beginning	0.62***	1			
Change in sleep problems	−0.26***	0.11	1		
Change in job demands	−0.22***	0.19**	0.25***	1	
Change in engaging leadership	0.14*	−0.09	−0.14*	−0.17*	1

**Note(s):** *p < 0.01, **p < 0.01, ***p < 0.001

**Source(s):** Authors’ own work

**Table 3.** Association between change in engaging leadership and recovery from work in the end of the study period. Results from linear regression analysis

	Step 1	Step 2	Step 3
Change in job demands	−0.266***	−0.24***	−0.24***
Change in engaging leadership		0.14**	0.14**
Change in job demands*change in engaging leadership			−0.00 (0.976)
*F* change	38.72***	10.28**	0.00 (0.976)
Adj. *R*^2^ change	0.58	0.02	0.00

**Note(s):** *p < 0.01, **p < 0.01, ***p < 0.001. Models are adjusted for recovery at the baseline, gender, age, preparing in a fragmented region, distance of response months and sleep problems. Table presents standardized regression coefficients and their statistical significance (*p*-values)

**Source(s):** Authors’ own work

## Discussion

Engaging leadership and recovery from work decreased among preparers of health-care and social services reform during the study period. Furthermore, change in engaging leadership was mild and positively associated with work recovery, giving some support to the first study hypothesis. We found no evidence supporting *H2*: engaging leadership style did not alleviate the negative effects of job demands on work recovery.

Experiences of engaging leadership declined especially among women, oldest preparers and those working in the fragmented regions. Fragmented regions were those, where coordination of the services in the beginning of the preparation started from scratch without previous cooperation with other health-care and social service organizations. Thus, in these regions, the higher job demands (Laitinen *et al.*, 2023) potentially limited managers possibilities to be available for their team members, to be aware of and to effectively coordinate their team members’ strengths, which are crucial elements of the engaging leadership style (Schaufeli, 2021; Mäkelä *et al.*, 2024). Changes in engaging leadership has been previously associated with employee’s motivation, ability to pursue shared goals and involvement (Mazzetti and Schaufeli, 2022), suggesting that decreases in engaging leadership may have had negative implications also for the whole success of the preparation of health-care and social services reform. More research, however, in this regard is needed in the future. From a practical point of view, results imply that in the future health-care and social services reforms, more attention should be paid also to management and to ensure that supervisors have time to be present for their subordinates and support their strengths regardless of the job demands.

Our findings suggest that adopting an engaging leadership style may aid work recovery, although the association was mild (*β* = 0.14, *p* = 0.002). To our knowledge, this is the first longitudinal study analyzing engaging leadership on work recovery during an organizational health-care and social services reform. Previously, Selander *et al.* (2023) discovered in a cross-sectional study that engaging leadership enhances work recovery among Finnish health-care and social service workers. Czakert *et al.* (2024) also found that transformational leadership, concept closely related to engaging leadership (Schaufeli, 2021), improves role clarity and, as a result, work recovery.

This study found no evidence to support *H2*, which stated that engaging leadership would alleviate the negative effects of job demands on work recovery. This contradicts a previous study by Selander *et al.* (2023), which discovered that engaging leadership style alleviates the negative impact of job strain on work recovery. Different measurements are one possible explanation for contradictory findings. In this study, we only considered job demands, whereas the previous study examined the imbalance between job demands and resources. Based on these findings, it is possible that engaging leadership style is more effective in fostering job resources such as feelings of autonomy, opportunities to use skills and experiences of meaningful work (Schaufeli, 2015, 2021) that are important determinants also in the daily work recovery process (Newman *et al.*, 2014; Virtanen *et al.*, 2021). Engaging leadership alone, however, is not sufficient in managing job demands as like has been suggested in the previous engaging leadership literature (Schaufeli, 2015). Thus, managing job demands calls for different approaches to leadership. Indeed, this study found that changes in job demands (*β* = 0.24, *p* < 0.001) had the strongest association with endpoint work recovery. Therefore, in the forthcoming implementations of the health-care and social services reforms, it is essential to place more emphasis on managing job demands. This requires contributions from all levels of the organization, including upper management, human resources professionals, supervisors and employees (Smollan, 2017). Effective management of job demands requires, for example, clearly defined work tasks and providing forums for open discussion on how to prioritize and limit work duties (Sallinen *et al.*, 2024).

### Strengths and limitations

The study’s main strength is its novelty: it expands previous work recovery literature by demonstrating that engaging leadership style can improve work recovery during health and social care reforms. Furthermore, it provides implications for work recovery among preparers of a major organizational reform, a group that, to our knowledge, has not previously been studied in organizational change research despite their critical role in the success of such changes. In addition, the extensive coverage of preparers from nearly all regions involved in health-care and social services reform in Finland enhances the generalizability of the findings within the Finnish context. More research about engaging leadership and work recovery during major organizational reforms is needed in the future, particularly in other countries.

The insights provided by this study needs to be acknowledged with limitations. First, the study relied on self-reported data and abbreviated measurements. Subjective evaluations imply that employees’ assessments of work recovery may influence their assessments of the level of engaging leadership, potentially leading to common-method variance bias in the data. In addition, the use of abbreviated scales reduces comparability with previous studies. However, abbreviated scales reduce response burden, making them more feasible to participants, which is especially important in large job well-being surveys such as this one. Furthermore, studies have demonstrated that the single-item work recovery (Kinnunen *et al.*, 2011) and ultra-short work engagement (Schaufeli *et al.*, 2019) scales assess the same underlying concepts as their longer counterparts. Second, the response rate and the number of respondents were low, limiting the findings generalizability to all preparers of the reform. Respondents also changed during the study period, probably because of workload and because regions were in various stages of the organizational reform when the survey began. In addition, combining monthly surveys may have resulted in the loss of some of the information from the follow-up design. Third, in this study, we only controlled for sleep problems because they were deemed the most important lifestyle variable influencing recovery (Sonnentag, 2018). However, there are other lifestyle variables that have an impact on work recovery and potentially the association between engaging leadership and work recovery. Thus, future studies should aim to include a broader range of lifestyle variables in the analysis. Finally, although we focused on the associations between engaging leadership and work recovery in this study, future studies should investigate also the impact of other leadership styles on work recovery, as well as whether leader’s own example of health behavior (Kranabetter and Niessen, 2017) and detachment from work (Sonnentag and Schiffner, 2019) have a positive impact on their follower’s recovery in their spare time during organizational changes.

## Conclusions

During the preparation of the major health-care and social services reform in Finland preparers experiences of engaging leadership and work recovery decreased. Good work recovery is a way to alleviate the negative effects of stressful work, and thus is an important resource for employees in a hectic work situation. This study showed that although engaging leadership could improve work recovery, it only had a minor effect on recovery and it was unable to alleviate the negative effect of job demands on work recovery. In preparation of future organizational reforms, controlling job demands is essential. Engaging leadership style should also be acknowledged and promoted alongside with managing job demands but also other leadership styles should be considered. More research of other aspects of leadership and their association with work recovery is needed.
